# The extent of late gadolinium enhancement can be assessed fast and reproducible using semi-quantitative method in patients with hypertrophic cardiomyopathy

**DOI:** 10.1186/1532-429X-15-S1-E118

**Published:** 2013-01-30

**Authors:** Frank Gommans, Jeannette Bakker, Etienne Cramer, Maurice J Kurvers, Freek W Verheugt, Marc A Brouwer, Marcel Kofflard

**Affiliations:** 1 Cardiology, Radboud University Nijmegen Medical Centre, Nijmegen, Netherlands; 2 Cardiology, Albert Schweitzer Hospital, Dordrecht, Netherlands; 3 Radiology, Albert Schweitzer Hospital, Dordrecht, Netherlands

## Background

In hypertrophic cardiomyopathy (HCM) assessment of late gadolinium enhancement (LGE) is increasingly important as LGE has been demonstrated to be an adverse prognosticator. Manual delineation of LGE after visual assessment is labour-intensive. Automatic quantification is limited by its dependence on signal/noise ratio and varying definition of LGE. Our aim is to assess inter-individual agreement of a fast semi-quantitative method based on visual assessment for quantification of LGE in HCM, which is feasible for daily clinical practice.

## Methods

We included 88 patients (60 clinical HCM patients, 28 subclinical HCM mutation carriers); age 48.9±15.0 years; 42 males. MRI was performed with 3D-contrast-enhanced inversion recovery imaging. Each segment of the AHA-17-segment model was scored to determine extent of LGE (0=no LGE, 1=1-25%, 2=26-50%, 3=51-75%, 4=76-100%) by two independent observers. The score was summed and converted to a percentage of the maximum possible score (68) to estimate the extent of LGE relative to LV mass. LGE results were compared to assess inter-individual agreement on the presence and extent of LGE in all subjects and in subjects that were LGE positive according to both observers, respectively.

## Results

The agreement on the presence and the extent of LGE was good (kappa=0.768 and r=0.861, p<0.001, respectively). Regarding presence of LGE: in 10 of 88 patients (11%) there was disagreement on the presence of LGE. In 8 of these 10 subjects the proportion of LV mass with LGE was scored between 1/68 and 3/68 by the other observer (i.e. <5%). Regarding extent of LGE: a difference in extent of LGE was reported in 42 of 43 patients with LGE. However, the size of the difference between observers was less than 3 points (i.e. <5%) in 75% of subjects (32/43). A difference of >6 points (i.e. >10%) was only seen in 4/43 patients (10%).

## Conclusions

There is good agreement between two independent observers on the presence and extent of LGE in HCM using the fast semi-quantitative method. We suggest that this method based on visual assessment might be incorporated in future studies and clinical practice as it substantially speeds up the analysis.

## Funding

None.

